# Trichogenic-selenium nanoparticles enhance disease suppressive ability of *Trichoderma* against downy mildew disease caused by *Sclerospora graminicola* in pearl millet

**DOI:** 10.1038/s41598-017-02737-6

**Published:** 2017-06-01

**Authors:** Boregowda Nandini, Puttaswamy Hariprasad, Harischandra Sripathy Prakash, Hunthrike Shekar Shetty, Nagaraja Geetha

**Affiliations:** 1 Department of Studies in Biotechnology, University of Mysore, Manasagangotri, Mysuru, 570 006 Karnataka India; 2 Centre for Rural Development and Technology, Indian Institute of Technology Delhi, Hauz Khas, New Delhi 110016 India

## Abstract

*Trichoderma* spp. are well known biocontrol agents used against phytopathogens. In the present work *Trichoderma-*mediated Selenium nanoparticles (SeNPs) were synthesized and extent of downy mildew (DM) disease control in pearl millet (PM) was studied. Six species of *Trichoderma* namely, *T*. *asperellum*, *T*. *harzianum*, *T*. *atroviride*, *T*. *virens*, *T*. *longibrachiatum* and *T*. *brevicompactum* were evaluated in the form of culture filtrate (CF), cell lysate (CL) and crude cell wall (CW) to synthesize SeNPs. All these components produced SeNPs, but CF was significant than CL and CW. The size of SeNPs ranged from 49.5 to 312.5 nm with zeta potential of +3.3 mv to −200 mv. The nanoparticles suppressed the growth, sporulation and zoospore viability of *Sclerospora graminicola* and these biological activities were inversely proportional to the size of SeNPs. Under greenhouse conditions, application of SeNPs and *T*. *asperellum* together enhanced the early plant growth and suppressed DM incidence as compared to their individual application. This study demonstrated the ability of Trichogenic-SeNPs to suppress growth and proliferation of *S*. *graminicola*, the incitant of DM of PM and their activity is inversely proportional to size of nanoparticles.

## Introduction

Selenium (Se) is a naturally occurring mineral in soil and being absorbed and accumulated by plants thereby entering the food chain. In addition, contaminated water and air also act as sources of Se exposure. Selenium is an important micronutrient required by both plants and animals. It was earlier recognized as toxic until Schwarz and Foltz^1^ reported its vital function in living organisms. Selenocystin present in the active site of glutathione peroxidase removes free radicals in cells reducing the adverse effect on cell components^2, 3^. On the other hand, increased concentration of Se in biological system leads to various health disorders^4–8^.

Selenium toxicity varies depending on its concentration and chemical form and usually present in the order, sodium selenite > selenium sulfide > elemental selenium^9^. Selenium nanoparticles (SeNPs) are less toxic than Se-methylselenocystenine (SeMSC), but up-regulate phase 2 enzymes as efficiently as SeMSC thereby preventing liver damage^10^. Because of its semi-conducting property Se and SeNPs are widely used in photovoltaic cells, electric rectifiers, photographic exposure meters and xenography^11, 12^. SeNPs have excellent bioavailability, unique physicochemical characteristics with high surface to volume ratio and exhibit admirable biological activity such as anti-microbial^13–15^, anti-oxidant^16^, anti-cancerous^17^ and anti-inflammatory^18^, and exhibit minimum toxicity than other forms of Se^10, 19, 20^.

By realizing the advantage of SeNPs over their other chemical forms, there is an increasing interest to generate SeNPs with various functionality. Most of the production methods employed for SeNPs involve chemicals such as hydrazine, sodium ascorbate or glycol (reducing agents), oxidation methods and by providing harsh conditions^21–23^. These methods are expensive, environmentally hazardous and require special equipment^24^. There is increasing interest in the synthesis of SeNPs using green nanobiotechnology which include microorganisms and plants or their byproducts (Secondary metabolites, proteins/enzymes, and lipids) with assistance of various biotechnological tools^25, 26^. These methods are ecofriendly, cost effective, overcome toxic and harsh chemicals, and do not need high energy.

*Trichoderma* spp. are ubiquitous soil fungi grow on a wide range of organic substrates, essentially cellulosic materials and take part in nutrient recycling thereby improving soil health^27, 28^. Symbiotically/endophytic association of these fungi with plants and protect them from biotic and abiotic stresses^29, 30^. *Trichoderma* spp. thrives under varying environmental conditions because of their high adaptability to growth regulation, sporulation and secretion of lytic enzymes^31^. These fungi are also known to resist/tolerate most of the pesticides used in agriculture^32–34^. These reasons make this group of fungi useful for industrial applications and as biofertilizer/biopesticide to improve plant health and yield. The genus *Trichoderma* is also explored for the production of silver nanoparticles (AgNPs) as it produced most positive results^35–38^.

Even with the advent of superior agro-technologies, plant diseases still poses a major problem in food production and is a threat to future food security. *Sclerospora graminicola* is a major constraint in pearl millet (PM) [*Pennisetum glaucum* (L.) R. Br.] production causing downy mildew (DM) disease. Under severe conditions it causes an estimated annual yield loss of <€11 million in India^39, 40^. The availability of DM resistant PM cultivars are limited and their durability is always questioned because of emergence of virulent pathogens. With increasing area under hybrid cultivation since 1970s, the disease has become more severe by the evolution of new virulent pathotypes in response to new hybrid genotypes^41^. Substantial work has been reported to manage PM-DM disease using biocontrol agents. Even after screening a large number of biocontrol agents still chemical pesticides are dominating under field conditions. This may cause considerable damage to the environment and lead to pesticide resistance in DM pathogen. Hence, eco-friendly integrated disease management strategies are gaining prominence across the world.

The present study aimed to develop reliable protocol for the synthesis of *Trichoderma-*assisted SeNPs possessing higher anti-mildew and zoosporicidal activity and evaluate them against DM pathogen. As per our knowledge and according to so far published reports this is the first attempt made to manage oomycetes disease in plants using Trichogenic-SeNPs and *Trichoderma* spp. together.

## Results

### Biosynthesis and characterization of SeNPs

**Figure 1:**
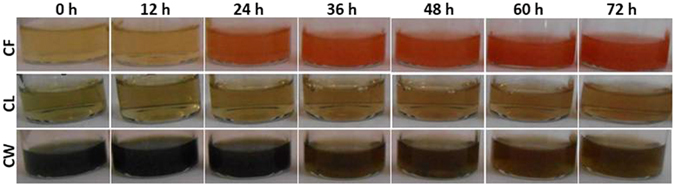
Trichogenesis of selenium nanoparticles (SeNPs) from the *T*. *asperellum* at different time intervals (hours). CW: Crude cell wall; CL: Cell lysate; CF: Culture filtrate.

The SeNPs were synthesized with 25 mM sodium selenite using culture filtrate (CF), cell lysate (CL) and crude cell wall (CW) of 6 different *Trichoderma* spp. [*T*. *asperellum* (T.as), *T*. *harzianum* (T.ha), *T*. *atroviride* (T.at), *T*. *virens* (T.vi), *T*. *longibrachiatum* (T.lo) and *T*. *brevicompactum* (T.br)]. Culture filtrate from *Trichoderma* spp. gave noticeable SeNPs production followed by CL and CW. In CF, formation of nanoparticles by the reduction of selenite ions could be visualized as change in solution color from pale yellow to insoluble orange-red within 12 h after incubation but in CL and CW, it was observed after 24 h (Fig. 1).

**Figure 2:**
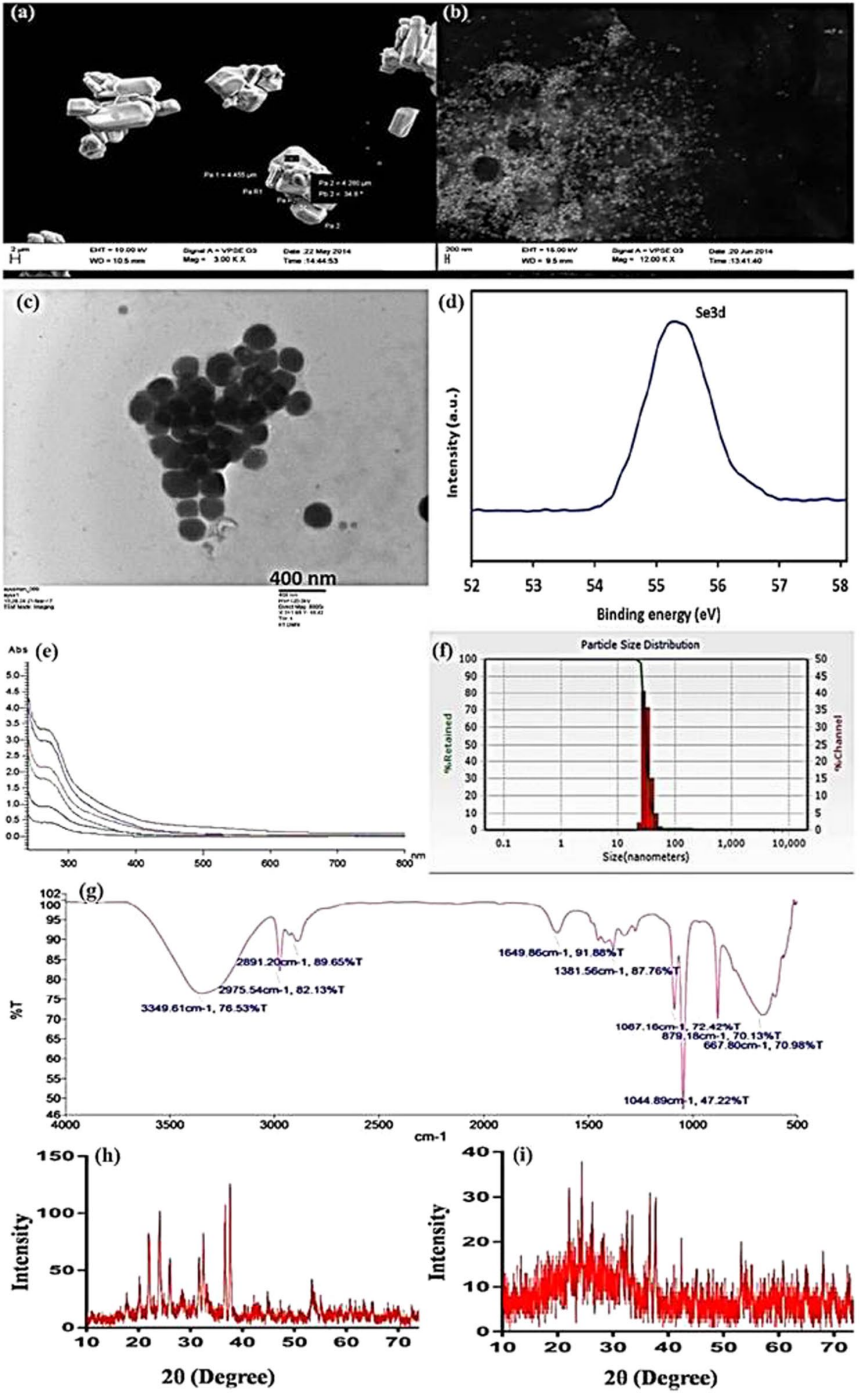
Characterization of Trichogenic-SeNPs generated using culture filtrate of *T*. *asperellum*. (**a**) Scanning electron microscopic (SEM) view of commercially available sodium selenite compound. (**b**) Scanning electron microscopic (SEM) view of Trichogenic-SeNPs. (**c**) Transmission electron microscopic (TEM) view of Trichogenic-SeNPs. (**d**) Se3d XPS spectrum. (**e**) UV- Visible spectrum showing the absorption peak at 280 nm. (**f**) Particle size distribution histogram of Trichogenic-SeNPs. (**g**) FTIR spectrum showing the reduction process with different % Transmittance. (**h**) XRD spectrum of the sodium selenite. (**i**) XRD spectrum showing the presence of Trichogenic-SeNPs.

SeNPs characters are presented in Table 1. Detailed characters of SeNPs generated from T.as-CF are shown in Fig. 2. Morphology and size of SeNPs were verified by SEM and TEM. Shapes of SeNPs were observed as hexagonal, near spherical and irregular (Fig. 2a,b,c, Supplementary Figs S1 and S2). X-ray Photoelectron spectroscopic (XPS) analysis of SeNPs was shown in Fig. 2d, an intense peak recorded at 55.6 eV which was corresponding to the binding energy of elemental Se. The characteristic red color of SeNPs observed in all reactions was due to the excitation of the surface plasmon vibrations of the monoclinic Se particles. The solutions of SeNPs when subjected to UV-visible spectral scan showed maximum absorption between 200 to 400 nm, corresponding to surface Plasmon resonance indicating the formation of SeNPs. In most of the CF-SeNPs, absorption peak appeared at 280 nm, a typical spectrum of T.as-CF-SeNPs is shown in Fig. 2e. In CL-SeNPs, an additional peak at 415 nm was present (Supplementary Fig. S3). The size of the nanoparticles generated from different methods varied from 49.5 nm (T.as-CF-SeNPs) to 312.5 nm (T.vi-CW-SeNPs). Zeta potential analysis of SeNPs obtained varied from +3.3 mv (T.at-CL-SeNPs) to −200 mv (T.as-CL-SeNPs) (Table 1).

**Table 1:** Summary of Trichogenic-selenium nanoparticles (SeNPs) characters.

Trichogenic-SeNPs	Size (nm)	Shape	Zeta potential/Polarity
**Culture filtrate**			
T.as-CF	49.5	Irregular	−63.8 mv/negative
T.ha-CF	60.8	Spherical	−14.4 mv/negative
T.vi-CF	96.2	Spherical	−28.2 mv/negative
T.lo-CF	87.5	Spherical	+11.8 mv/positive
T.at-CF	157.9	Irregular	−7.8 mv/negative
T.br-CF	99.6	Irregular	+7.3 mv/positive
**Cell lysate**			
T.as-CL	61.3	Irregular	−200 mv/negative
T.ha-CL	140.4	Spherical	+5.7 mv/positive
T.vi-CL	158.8	Irregular	+6.7 mv/positive
T.lo-CL	256.1	Irregular	−38.1 mv/negative
T.at-CL	168.4	Irregular	+3.3 mv/positive
T.br-CL	109.2	Irregular	+5.3 mv/positive
**Crude cell wall**			
T.as-CW	130.2	Spherical	−26.1 mv/negative
T.ha-CW	103.5	Spherical	−16.7 mv/negative
T.vi-CW	312.5	Irregular	−28.0 mv/negative
T.lo-CW	158.4	Irregular	−15.6 mv/negative
T.at-CW	67.9	Irregular	+6.9 mv/positive
T.br-CW	199.6	Irregular	+7.3 mv/positive

T.as – *T*. *asperellum*; T.ha – *T*. *harzianum*; T.vi – *T*. *virens*; T.lo – *T*. *longibrachiatum*; T.at – *T*. *atroviride*; T.br – *T*. *brevicompactum*; CF – Culture filtrate, CL – cell lysate; CW – crude cell wall.

FTIR analysis of T.as-CF-SeNPs confirmed the presence of elemental SeNPs, an indication of the reduction process (Fig. 2g). It disclosed a broad peak at 3349 cm^−1^, which is the trait of O-H stretching form and N-H stretch in amine group. The peaks at 2975 and 2891 cm^−1^ correspond to the asymmetric stretching vibration of –CH_3_, the asymmetric and symmetric stretching vibrations of –CH_2_, respectively. The peaks, at 1649 and 1381 cm^−1^ specify the incidence of interaction between C=O and C–N groups and SeNPs separately. Hence, the association of proteins with SeNPs is confirmed and possibly they prevent agglomeration of particles by stabilizing SeNPs in the medium. The distinctive XRD patterns of commercially available sodium selenite and T.as-CF-SeNPsare presented in Fig. 2h and i, respectively. In Fig. 2i, amorphous/nano-crystalline nature of the synthesized SeNPs is known by noisier feature with wider peaks and each diffraction pattern peaks in the range of 2θ. In SeNPs, there are no clear sharp Bragg reflections which indicate the association of SeNPs with protein.

### Anti-mildew and zoosporicidal activity of SeNPs

**Figure 3:**
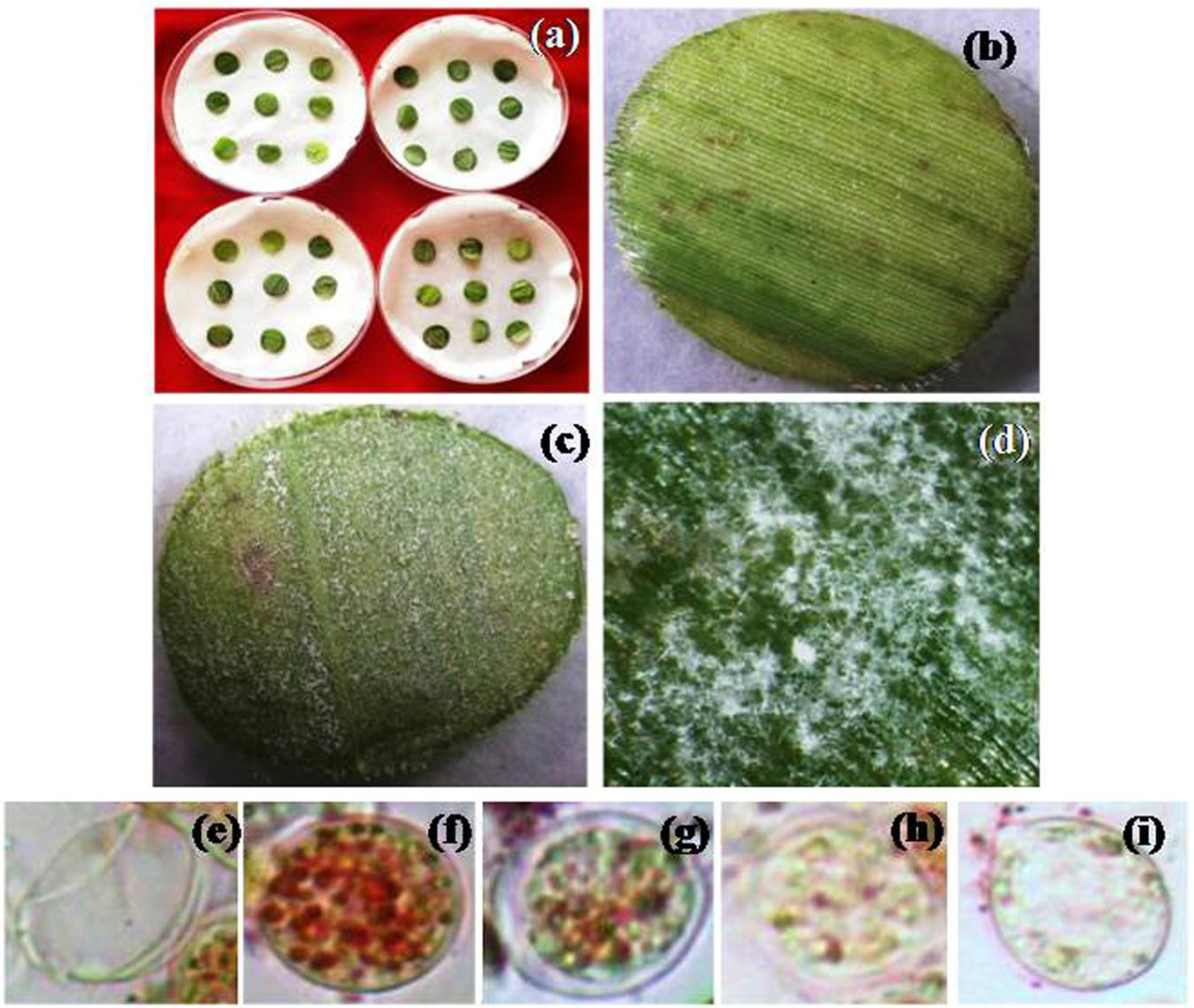
Anti-mildew activity. (**a**) Set up showing anti-mildew activity assay. (**b**) T.as-CF-SeNPs treatment suppressed the growth and sporulation of *Sclerosporagraminicola*. (**c**) Untreated infected leaf showing profuse sporulation of *Sclerospora graminicola*. (**d**) Close up view of infected leaf showing sporangia and sporangiospores, observed under stereo binocular microscope. (**e**–**i**) Viable and non-viable zoospores observed under microscope after TTC staining. (**e**) Empty sporangia after releasing viable zoospores (control), (**f**). Deeply red colored zoospores in sporangia (control), (**g**). T.ha-CF-SeNPs treatment showing mixture of live and dead zoospores, (**h**) and (**i**) zoospores showing reduction in viability of zoospores in T.br-CF-SeNPs and T.as-CF-SeNPs treatment, respectively.

**Figure 4:**
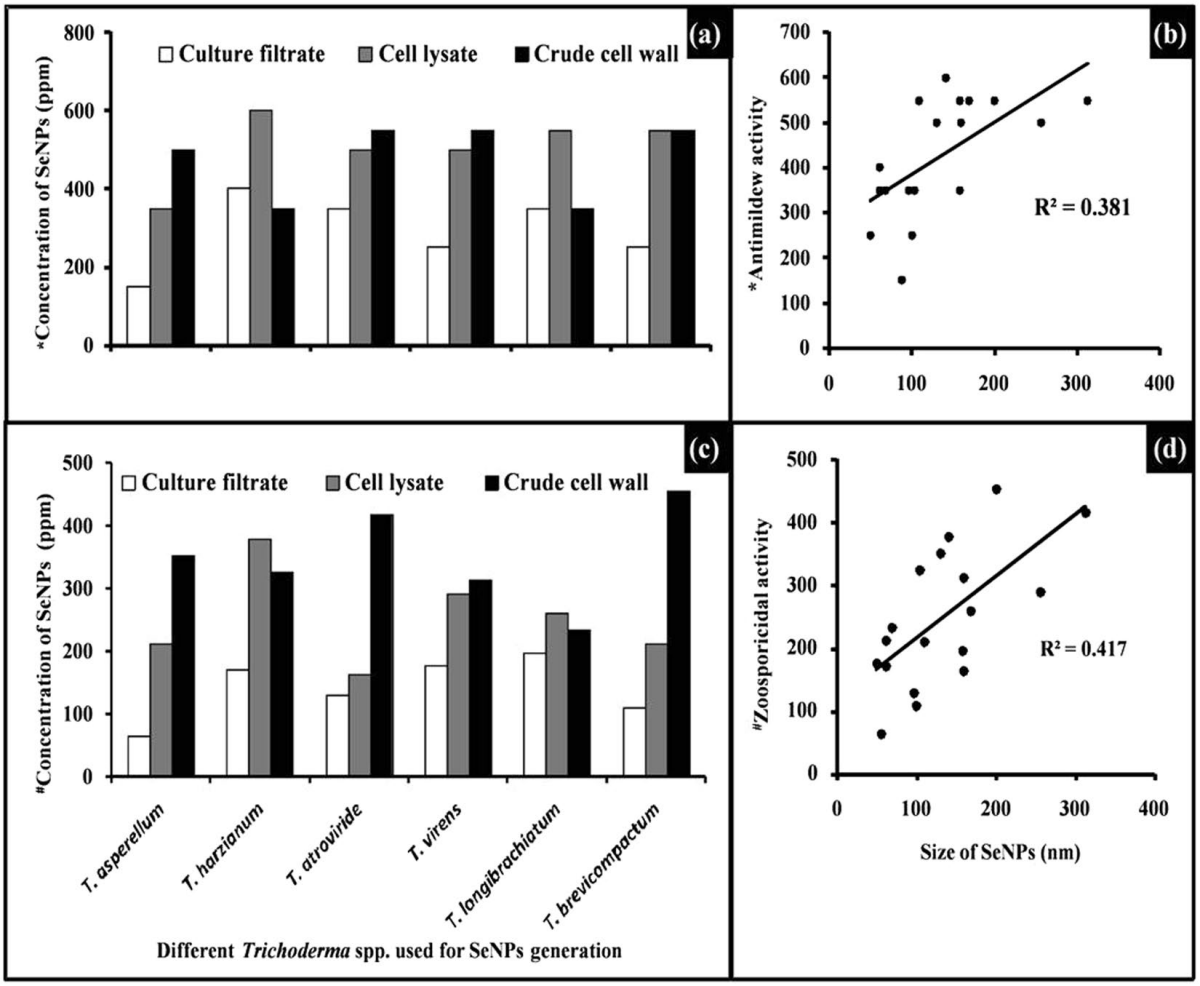
Anti-mildew and zoosporicidal activity of Trichogenic-SeNPs. (**a**) Anti-mildew activity of Trichogenic-SeNPs generated using CF, CL and CW of *Trichoderma* spp. (**b**) Correlation between anti-mildew activity and size of SeNPs. (**c**) Zoosporicidal activity of Trichogenic-SeNPs generated using CF, CL and CW. (**d**) Correlation between zoosporicidal activity and size of SeNPs. *Minimum concentration SeNPs showing complete inhibition of sporulation. ^**#**^Concentration of SeNPs corresponding to IC_50_ value. Valuesare means of three replicates.

Selenium nanoparticles suppressed sporulation of *S*. *graminicola* when applied on to the surface of infected PM leaf. The extent of suppression varied significantly (*P* ≤ 0.05) among the SeNPs generated by different methods. CF-SeNPs significantly (*P* ≤ 0.05) suppressed sporulation when compared to CL-SeNPs and CW-SeNPs (Fig. 3a–d). Maximum suppression of sporulation was recorded with SeNPs generated by T.as-CF followed by T.br-CF as indicated by the low minimum inhibitory concentration (MIC) value of 150 ppm and 250 ppm, respectively (Fig. 4a, Table S1).

This trend was also observed with SeNPs, when subjected to zoosporicidal assay. CF-SeNPs of different *Trichoderma* showed significantly (*P* ≤ 0.05) lower IC_50_ values than SeNPs generated using CL and CW. Least IC_50_ value of 64 ppm and 109 ppm were recorded for T.as-CF-SeNPs and T.br-CF-SeNPs, respectively (Fig. 4c). Microscopic observation of sporangia revealed that T.as-CF-SeNPs reduced viability of sporangia significantly (*P* ≤ 0.05), whereas in control the sporangia were red colored due to accumulation of insoluble formazan (Fig. 3e–i).

It was also evident from the assays that the size of SeNPs is inversely proportional to its DM pathogen suppressive ability (Fig. 4b and d).

### Effect of seed treatment with combined T.as-CF-SeNPs and *Trichoderma* on plant growth and disease protection under greenhouse conditions

Seed treatment with SeNPs (100 ppm) promoted all the plant growth parameters under greenhouse conditions as compared to control. All *Trichoderma*-SeNPs in combination significantly (*P* ≤ 0.05) increased plant height, dry weight, number of tillers per plant and chlorophyll content over that of control (Table 2). Foliar spray with SeNPs (100 ppm) did not affect any of the plant growth parameters and has not visually shown any phytotoxic effect. Among different treatments, application of *T*. *asperelleum* + SeNPs significant (*P* ≤ 0.05) improved plant height (24.9 cm), number of tillers (3.40 tillers/seedling) and chlorophyll content (3.76 mg/g) of PM seedlings, as compared to control plants (Table 2).

**Table 2:** Effect of combined application of *Trichoderma* and T.as-CF-SeNPs on growth parameters and downy mildew disease incidence in pearl millet.

Treatments			APH (cm)	Number of tillers/seedling	Dry weight (g/seedling)	Total Chlorophyll (mg/g fresh weight)	Disease Incidence (%)
Seed treament	SeNPs Foliar Sparay	Challenge inoculation					
Control	−	−	20.41 ± 0.55^ab^	2.68 ± 0.15^defgh^	2.43 ± 0.06^fg^	3.63 ± 0.10^a^	—
	+		20.83 ± 0.89^ab^	2.60 ± 0.10^gh^	2.43 ± 0.07^fg^	3.55 ± 0.13^a^	—
	−	+	11.50 ± 0.48^d^	2.10 ± 0.15^i^	1.15 ± 0.03^i^	2.28 ± 0.10^b^	93 ± 1.45^a^
	+		15.35 ± 1.18^cd^	2.45 ± 0.09^hi^	1.65 ± 0.06^h^	2.95 ± 0.04^ab^	49 ± 1.45^b^
T.as + SeNPs	−	−	24.71 ± 0.62^a^	3.22 ± 0.06^ab^	3.03 ± 0.06^abc^	3.64 ± 0.10^a^	—
	+		24.85 ± 1.41^a^	3.40 ± 0.06^a^	3.04 ± 0.09^ab^	3.76 ± 0.08^a^	—
	−	+	21.60 ± 0.72^ab^	2.88 ± 0.09^bcdefgh^	2.64 ± 0.07^defg^	3.53 ± 0.19^a^	20 ± 0.88^d^
	+		23.55 ± 1.19^a^	3.14 ± 0.08^abc^	2.66 ± 0.08^bcedf^	3.64 ± 0.06^a^	12 ± 1.15^e^
T.ha + SeNPs	−	−	22.02 ± 0.76^ab^	3.13 ± 0.04^abcd^	2.93 ± 0.06^abcd^	3.62 ± 0.10^a^	—
	+		22.39 ± 0.65^ab^	3.23 ± 0.09^ab^	2.93 ± 0.06^abcde^	3.66 ± 0.12^a^	—
	−	+	21.35 ± 0.61^ab^	2.90 ± 0.06^bcdefg^	2.54 ± 0.07^efg^	3.32 ± 0.13^a^	29 ± 1.20^c^
	+		21.54 ± 0.38^ab^	3.12 ± 0.01^abcd^	2.26 ± 0.04^g^	3.51 ± 0.14^a^	14 ± 0.88^e^
T.at + SeNPs	−	−	20.95 ± 1.41^ab^	3.08 ± 0.06^abcde^	2.83 ± 0.07^abcde^	3.59 ± 0.26^a^	—
	+		20.57 ± 0.56^ab^	3.00 ± 0.06^abcdefg^	2.88 ± 0.09^abcde^	3.53 ± 0.17^a^	—
	−	+	18.72 ± 0.23^bc^	2.63 ± 0.08^fgh^	2.75 ± 0.09^abcdef^	3.16 ± 0.14^a^	30 ± 2.40^c^
	+		20.56 ± 1.18^ab^	2.96 ± 0.09^abcdefg^	2.86 ± 0.09^abcde^	3.50 ± 0.12^a^	21 ± 1.53^d^
T.vi + SeNPs	−	−	21.44 ± 0.48^ab^	3.03 ± 0.12^abcdefg^	2.94 ± 0.07^abcd^	3.72 ± 0.06^a^	—
	+		21.05 ± 1.04^ab^	3.05 ± 0.09^abcdef^	2.93 ± 0.12^abcde^	3.66 ± 0.08^a^	—
	−	+	18.43 ± 0.54^bc^	2.75 ± 0.09^cdefgh^	2.82 ± 0.08^abcde^	3.27 ± 0.05^a^	28 ± 0.58^c^
	+		20.90 ± 1.42^ab^	2.91 ± 0.06^bcdefg^	2.89 ± 0.10^abcde^	3.52 ± 0.16^a^	25 ± 1.45^cd^
T.lo + SeNPs	−	−	22.92 ± 0.57^ab^	3.10 ± 0.06^abcde^	2.84 ± 0.05^abcde^	3.72 ± 0.12^a^	—
	+		23.18 ± 0.98^ab^	3.03 ± 0.08^abcdefg^	3.05 ± 0.08^a^	3.60 ± 0.25^a^	—
	−	+	21.66 ± 0.46^ab^	2.66 ± 0.03^efgh^	2.82 ± 0.07^abcdef^	3.46 ± 0.26^a^	26 ± 0.88^cd^
	+		22.57 ± 0.66^ab^	2.80 ± 0.06^bcdefgh^	2.83 ± 0.07^abcde^	3.70 ± 0.10^a^	22 ± 1.45^d^
T.br + SeNPs	−	−	21.72 ± 0.49^ab^	3.00 ± 0.06^abcdefg^	2.82 ± 0.07^abcde^	3.73 ± 0.13^a^	—
	+		21.26 ± 1.39^ab^	3.03 ± 0.09^abcdefg^	2.85 ± 0.05^abcde^	3.58 ± 0.21^a^	—
	−	+	20.49 ± 0.64^ab^	2.81 ± 0.06^bcdefgh^	2.65 ± 0.06^cdef^	3.43 ± 0.07^a^	21 ± 0.58^d^
	+		21.28 ± 0.90^ab^	2.92 ± 0.06^bcdefg^	2.82 ± 0.06^abcde^	3.70 ± 0.15^a^	18 ± 0.88^de^
Apron	−	−	20.27 ± 0.61^ab^	2.71 ± 0.06^cdefgh^	2.65 ± 0.05^cdefg^	3.66 ± 0.13^a^	—
	−	+	20.55 ± 0.59^ab^	2.70 ± 0.06^cdefgh^	2.68 ± 0.05^abcdef^	3.60 ± 0.25^a^	9 ± 0.88^e^

APH - average plant height; T.as - *T*. *asperellum*; T.ha – *T*. *harzianum*; T.vi – *T*. *virens*; T.lo – *T*. *longibrachiatum*; T.at – *T*. *atroviride*; T.br – *T*. *brevicompactum*. Values are the mean of three replicates; values within column sharing the same letter(s) are not significantly different according to Tukey’s HSD at *P* ≤ 0.05.

All combinations of *Trichoderma* spp. and SeNPs suppressed DM disease of PM under greenhouse conditions as compared to its individual application and control. The extent of disease control varied considerably depending upon combinations. Both individual and combined applications significantly (*P* ≤ 0.05) reduced DM infected plants as compared with their respective controls. Disease protection ability of all *Trichoderma* spp. increased in the presence of SeNPs. Further enhancement in disease protection efficacy of the combination was observed, when additional SeNPs was applied as spray treatment. Maximum protection was observed with *T*. *asperellum* + SeNPs applied as seed treatment followed by foliar spray with SeNPs, which recorded 12% disease incidence as compared to 87% in control (Table 2). The control challenged plants recorded significant (*P* ≤ 0.05) reduction in plant height (11.5 cm), number of tillers (2.10 tillers/seedling) and chlorophyll content (2.28 mg/g).

## Discussion

In this study, SeNPs generated from CF, CL and CW of six *Trichoderma* spp. were evaluated to suppress DM disease in PM in combination with *Trichoderma* spp. The CF of all the *Trichoderma* spp. were best in producing SeNPs and it was visualized as early as 12 h after incubation in comparison with CL and CW.

Use of a cellular component of microbes is always considered as more advantageous in generating nanoparticles, than using whole microorganism. When live microorganisms are used, control over the size of nanoparticles cannot be achieved as growth stage and incubation period greatly affect the size and characters of nanoparticles^42–44^. Also,use of culture filtrate makes the downstream process easier. Culture filtrates of *Trichoderma* spp.^35, 38^, *Fusarium oxysporum* ^45^, *Aspergillus flavus* ^46^ and *Aspergillus terreus* ^47^ were successfully used earlier for the generation of AgNPs. Similarly, Zare *et al*.^48^ by using culture filtrate of *A*. *terreus* generated spherical shaped SeNPs with an average size of 47 nm.

Previous studies, have established that biogenesis of NPs depends on several factors including, pH, temperature, raw material form and concentration, incubation period, cocktail of the biochemical present in the biological extracts^49–53^. Characterization of 18 SeNPs generated from three components of six *Trichoderma* spp. had common features such as brick red color of solutions and maximum absorbance at 200–400 nm. Results of XPS analysis confirmed the presence of elemental Se. FTIR, XRD pattern, size and shape as observed under TEM and SEM and zeta potential were not same among the SeNPs generated through different methods. Characters SeNPs generated in our studies are in accordance with previous reports^15, 53–58^. The present results clearly indicate the differential ability of *Trichoderma* to generate SeNPs. Such observation was also made by Devi *et al*.^35^, when they tested 75 isolates of *Trichoderma* spp. belonging to five different species for AgNPs generation. In our studies, CF of *T*. *asperellum* yielded the most favourable results with respect to the incubation period, zeta potential and size of the SeNPs.

In our experiments, the most striking difference observed between the SeNPs was their differential ability to suppress the sporulation of DM pathogen on PM leaves and their zoosporicidal activity. These phenomena of SeNPs may be due to its variable characters as discussed earlier. Recently, Ajitha *et al*.^59^ reported the dependency of AgNPs antimicrobial activity on their particle size. They observed that decrease in the size of AgNPs increased the antimicrobial activity against *E*. *coli*, *Pseudomonas* spp. *Aspergillus niger* and *Penicillium* spp. Studies on shape dependent activity of AgNPs with *E*. *coli* made by Pal *et al*.^60^ revealed that, truncated triangular AgNPs were remarkably inhibited bacterial growth as compared to spherical and rod-shaped AgNPs. It was observed that the anti-mildew and zoosporicidal activity of SeNPs was inversely proportional to its size (Fig. 4b and d).

The application of nanometals in plant disease management is promising as an alternative to chemical pesticides. DNA-directed AgNPs grown on graphine oxide (Go) suppresses bacterial spot of tomato caused by *Xanthomonas perforans* ^61^ and powdery mildew in cucrubits when applied as foliar spray at 100 ppm concentration^62^. Jo *et al*.^63^ evaluated the antifungal activity of silver ions and silver nanoparticles against two plant pathogenic fungi, *Bipolaris sorkiniana* and *Mangoporthe grisea*. Foliar application of AgNPs prior to the application of pathogens suppressed fungal growth and reduced disease incidence in perennial ray grass. But the use of SeNPs for plant disease management is not reported.

Anti-mildew activity revealed that highest concentration (1000 ppm) of SeNPs treatment was not phytotoxic to PM leaves. At 100 ppm concentration SeNPs did not inhibit the growth of *Trichoderma* spp. and PM seedlings (Supplementary Fig. S4). The sub-inhibitory concentration of SeNPs stimulates the growth of *A*. *niger* which could be attributed to the microbial growth promotion by trace elements^64^. Also low concentrations of Se improve plant growth and yield of Brassica, ryegrass, potato and soybean^65–68^. In accordance with previous reports, at lower concentration SeNPs applied as seed treatment along with *Trichoderma* enhances plant growth parameters.

## Materials and Methods

### Microorganisms and culture conditions

#### Trichoderma spp

All *Trichoderma* spp. [*T*. *asperellum* (DL-81: KM100835), *T*. *harzianum* (HR-73: KM100834), *T*. *atroviride* (MH-50: KM100830), *T*. *virens* (MP-60: KM100832), *T*. *longibrachiatum* (MP-59: KM100831) and *T*. *brevicompactum* (UP-91: KM100836)] used in the study were collected from the culture collection of Department of Biotechnology, University of Mysore. These fungi were originally isolated from PM rhizosphere soil samples and were well characterized individually for plant growth promoting and downy mildew (DM) disease suppressing ability (unpublished data). All the fungi were subcultured once in 15 days and maintained on Potato dextrose agar (PDA) at 28 ± 1 °C throughout the experimental period.

For biosynthesis of SeNPs, the test fungi were grown on Potato dextrose broth (PDB) under dark and static conditions at 28 ± 1 °C for 7 days. At the end of incubation period, mycelial mat was collected by filtering through four layers of muslin cloth and washed three times with sterile distilled water. Crude cell wall debris was washed three times with sterile distilled water before further use. The culture filtrates (CF), cell lysate (CL) and crude cell wall (CW) from six *Trichoderma* spp. were used for SeNPs production. Three g of wet biomass of mycelial mat was ground to fine powder with liquid nitrogen and suspended in 25 ml sterile distilled water, further the cells were disrupted by sonication (8 × 8 s, 18 micron amplitude, 30 s cooling on ice between sonication cycles, Sonic Vibra cell, Sonic and Materials Inc., USA)^69^. To ensure complete lysis, 5 μl of the solution was spread over Potato dextrose agar (PDA) and analyzed for fungal growth upto five days. No fungal colonies on PDA indicated the complete disruption of the fungal mycelia. The lysed mycelial solution was centrifuged at 12000 rpm for 10 min at 4 °C and crude cell wall debris (pellet) was separated from cell lysate (supernatant).

For greenhouse studies, all *Trichoderma* spp. were grown on PDA for 12 days on 90 mm Petri plates at 28 ± 1 °C. At the end of incubation period, the conidia were dislodged by adding 5 ml of sterile distilled water and by using a sterile soft brush. The conidial suspension was washed three times with sterile distilled water, the final concentration adjusted to 1 × 10^8^ conidia/ml using hemocytometer and used for further experiments.

#### Sclerospora graminicola

Downy mildew (DM) pathogen, *S*. *graminicola* sick plot is maintained at the Department of Biotechnology, University of Mysore, Mysuru (N24°18′, E79°26′, 903 m altitude) since last three decades under All India co-ordinated pearl millet improvement project (AICPMIP).

The infected leaves showing typical DM disease symptoms were collected from field grown PM cv. 7042S in the evening hours. The collected leaves showing profuse DM growth on the abaxial surface of leaves were washed under running tap water to remove old sporulation and adhering extraneous particles. The leaves were blot dried and incubated overnight in a humid chamber (>70% RH and 20 °C) by keeping abaxial surface upwards. Next day early morning the sporangia grown profusely on abaxial leaf surface were collected in distilled water using a sterile soft brush without damaging sporangia. Immediately, the final concentration was adjusted to 5 × 10^3^ sporangia/ml using hemocytometer and used as inoculum.

### Biosynthesis of SeNPs

Culture filtrate, CL and CW components were used for SeNPs biosynthesis. Initiation of SeNPs synthesis was done by adding 20 ml CF or CL or 3 g wet weight of CW to 70 ml of sterile distilled water containing 25 mM sodium selenite made up to 100 ml using sodium selenite solution (25 mM)^35^. The reaction mixture was kept at 28 ± 1 °C on a shaker at 150 rpm for 6 days. The formation of nanoparticles was first visually examined for change in color of reaction mixture. At different time intervals of the reaction, the reaction mixture was collected and the nanoparticles were precipitated by centrifuging at 10,000 rpm for 10 min. The precipitate was washed with double distilled water and further purified as explained by Zhang *et al*.^70^. SeNPs from CW was extracted by the method of Sonkusre *et al*.^71^. Throughout the experimental period, appropriate controls were maintained.

### Characterization of biosynthesized SeNPs

Ultraviolet-visible spectrum from 200 to 800 nm was recorded at the resolution of 1 nm in HITACHI U-200 Spectrophotometer. X-ray photoelectron spectroscopy (XPS) measurements were carried out on an SPECS system using an Mg Ka x-ray source. The system was calibrated with C1s peak (284.8 eV). The functional association of SeNPs was analyzed by FTIR spectrum (PerkinElmer Spectrum NIOS2) after grinding with KBr. The spectrum was recorded at a resolution of 4 cm^−1^ at the range of 500–4000 cm^−1^. X-ray diffraction (XRD) pattern of the samples was generated using εMMA X-Ray Diffractometer (Braeside, Australia) operating at a voltage of 40 kV and current of 20 mA. The scanning was done in the 2θ range of 20° to 80° at 0.02°/min with a time constant of 2 sec. The size and morphology of Trichogenic-SeNPs were assessed by scanning electron microscopy (SEM) (Zeiss EVOLS 15) and Transmission electron microscopy (TEM) by coating them on a thin film of carbon-coated copper grid. Zeta potential and particle distributions of SeNPs were detected using Microtrac SL-PS-25 Rev.

### Anti-mildew activity

Leaf disc method was followed to assess the anti-mildew activity of the synthesized SeNPs against DM pathogen of PM following the method of Girijamba *et al*.^72^. The diseased leaves were collected from the sick plot and washed with distilled water and blot-dried to remove excess water. Leaf discs of 10 mm were obtained by cutting the infected leaf using sterile cork borer. The discs were treated with 0 to 1000 ppm of SeNPs at an interval of 50 ppm for 5 min. The treated and control leaf discs were placed (adaxial surface downwards) on the moist blotter paper in petridish and the plates were incubated for 12–14 h in moist chambers (>70% RH and 20 °C) under dark condition. The treated leaf discs were observed for sporulation under a stereobinocular microscope. Various sporulation behaviors in leaves were recorded on the basis of inhibition scale: A (100% inhibition), B (<100–75> % inhibition), C (<75–50> % inhibition), D (<50–25> % inhibition) and D (<25–00> % inhibition) (Table S1) and minimum concentration of SeNPs needed for 100% suppression of sporulation was recorded.

### Zoosporicidal assay

Fresh sporangia were harvested from the infected leaves as explained earlier. The sporangial concentration was immediately adjusted to 5 × 10^3^/ml using hemocytometer and it was used as inoculum. The reaction mixture containing different concentrations (0 to 1000 ppm at an interval of 50 ppm) of SeNPs (100 µl) and inoculum (100 µl) was incubated in dark for one h. After incubation, 20 µl of Triphenyltetrazolium chloride (TTC) solution were added and further incubated for 30 min. The sporangia were observed under compound microscope for the red colored insoluble formazan. The resultant mixture was centrifuged at 8000 rpm for 8 min and the pellet was washed three times with distilled water. The pellet was mixed with 1 ml of 95% ethanol and incubated in a waterbath at 85 °C for 30 min. The mixture was centrifuged at 8000 rpm for 8 min, 200 µl supernatant was transferred to a microtiter plate and read at 485 nm against respective controls. The percent inhibition was calculated by considering color developed in control as 100% viability. The assay was performed in triplicate for each treatment.

### Disease protection studies under greenhouse conditions

Greenhouse study was limited only to T.as-CF-SeNPs, since it showed better anti-mildew and zoosporicidal activity. The seeds of PM (cv. 7042 S), which are highly susceptible to DM disease was used after surface sterilization. A formulation containing different *Trichoderma* (1 × 10^8^ conidia/ml), T.as-CF-SeNPs (100ppm) and CMC (0.4%, W/V) was prepared in sterile water. The PM Seeds were treated with this formulation on a rotary shaker (200 rpm) for 30 min at 30 ± 1 °C. Treated seeds after separation from the solution by filtration were spread uniformly on three layers of blotter sheet and dried overnight in laminar air flow. The seeds treated with distilled water amended with CMC and Apron 35 SD (6 g/kg seeds) served as negative and positive control, respectively. The seeds were sown in earthen pots filled with sterilized potting mixture (2:1:1, soil:sand:farm yard manure, v/v). The seedlings were raised under greenhouse conditions (25 ± 2 °C, 80% RH, natural sunlight). To one set of experiment, an additional foliar spray with T. as-CF-SeNPs (100 ppm prepared in sterile distilled water) was given 24 h prior to challenge inoculation. Three-day-old seedlings were challenge inoculated whorally with zoospore suspension during early morning for three consecutive days^73^. Pots containing seedlings raised with different treatments followed by challenge-inoculation were arranged in a randomized complete block design and watered as and when required. Thirty days after sowing (DAS), plant height was measured from the base to the tip of the plant, seedlings were uprooted without damaging the root system washed under running tap water to remove adhering soil particles and blot dried. The dry weight of the seedlings was determined after drying in an oven at 60 °C until the constant weight was achieved. Chlorophyll content in leaves of different treatments was determined following the standard procedures^74^. Before growth parameter analysis, the plants showing typical DM symptoms such as, stunted growth, chlorosis and sporulation on the abaxial leaf surface were recorded and the percentage disease incidence was calculated. For each treatment, six pots were maintained in triplicates, each pot containing 6–8 seedlings. Each pot was labeled and arranged in a randomized order. The control plants (without challenge inoculation) were maintained separately to avoid cross contamination of pathogen. The whole experiment was replicated thrice and the data expressed as the mean of three experiments.

### Statistical analysis

The data collected from laboratory and greenhouse experiments were subjected to analysis of variance using SPSS Inc. 17.0. Significant treatment effects were determined by calculating *F* values (*P* ≤ 0.05). Treatment means were compared for significant differences (at *P* ≤ 0.05) using Tukey’s honest significant differences (HSD) test.

## Conclusion

The results suggest that Trichogenic-SeNPs are anti-mildew and zoosporicidal and the activity is inversely proportional to the size of SeNPs. The SeNPs generated using CF of *T*. *asperellum* had better anti-mildew and zoosporicidal activity and they are highly effective when applied in combination with different *Trichoderma* spp. in reducing DM disease in PM. This study points to the potential in integrated approach for management of DM disease in PM. This opens up a new avenue where *Trichoderma* formulations along with SeNPs can be successfully employed for plant disease management.

## Electronic supplementary material

Supplementary Information
