# Supplementary material for: Trichogenic-selenium nanoparticles enhance disease suppressive ability of Trichoderma against downy mildew disease caused by Sclerospora graminicola in pearl millet
Source: Sci Rep. 2017 Jun 1;7:2612. doi: 10.1038/s41598-017-02737-6 (PMC5453930; doi:10.1038/s41598-017-02737-6)
Supplement: Supplementary file 1 — Supplementary Information [file 41598_2017_2737_MOESM1_ESM.pdf]

Scientific Reports

Supplementary Information

Trichogenic-selenium nanoparticles enhance disease suppressive ability of *Trichoderma* against downy mildew disease caused by *Sclerospora graminicola* in pearl millet

Boregowda Nandini[1], Puttaswamy Hariprasad[2], Harischandra Sripathy Prakash[1], Hunthrike Shekar Shetty[1], Nagaraja Geetha[1,*]

Affiliations

[1] Department of Studies in Biotechnology, University of Mysore, Manasagangotri, Mysuru – 570 006, Karnataka, India

[2]Centre for Rural Development and Technology, Indian Institute of Technology Delhi, HauzKhas, New Delhi 110016, India.

Corresponding author*

Dr. Geetha N,
Department of Studies in Biotechnology,
Manasagangotri, University of Mysore,
Mysuru-570006.
E-mail: geetha@appbot.uni-mysore.ac.in
Telephone: 0821-2419462; Mobile no: 9611090434

Figure legends

Fig S1. Scanning electron microscopic (SEM) view of Trichogenic-SeNPs of different *Trichoderma* used in the present study

Fig S2. Transmission electron microscopic (TEM) images of Trichogenic-SeNPs of different *Trichoderma* used in the present study.

Fig S3. UV - Visible spectrum of CL-SeNPs showing an additional peak absorption peak at 415 nm.

Fig S4. Effect of Trichogenic-SeNPs on the growth of (a) *T. asperellum* (b) pearl millet (PM) at 100 ppm concentration SeNPs.

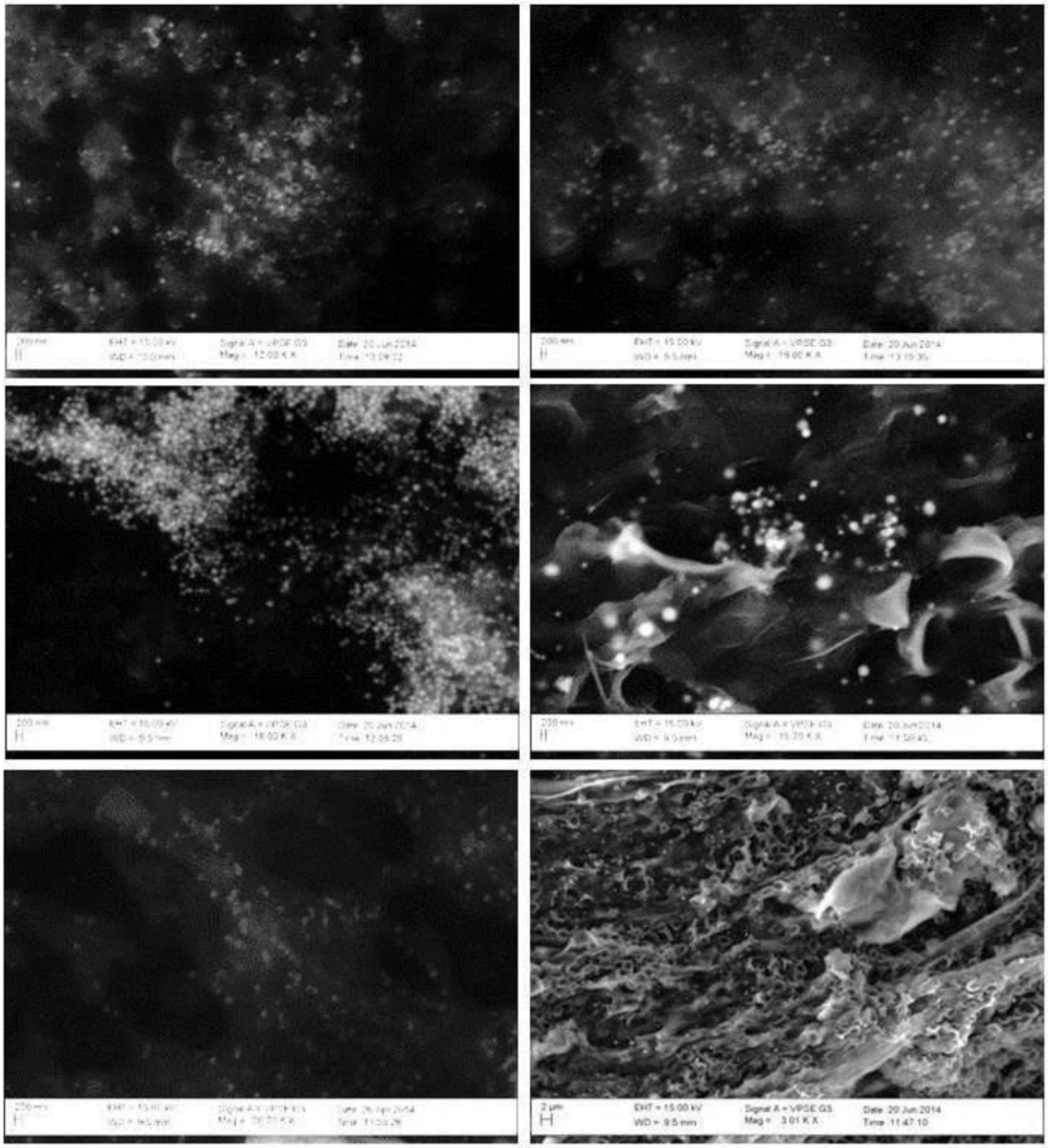

Fig S1. Scanning electron microscopic (SEM) images of Trichogenic-SeNPs of different *Trichoderma* used in the present study.

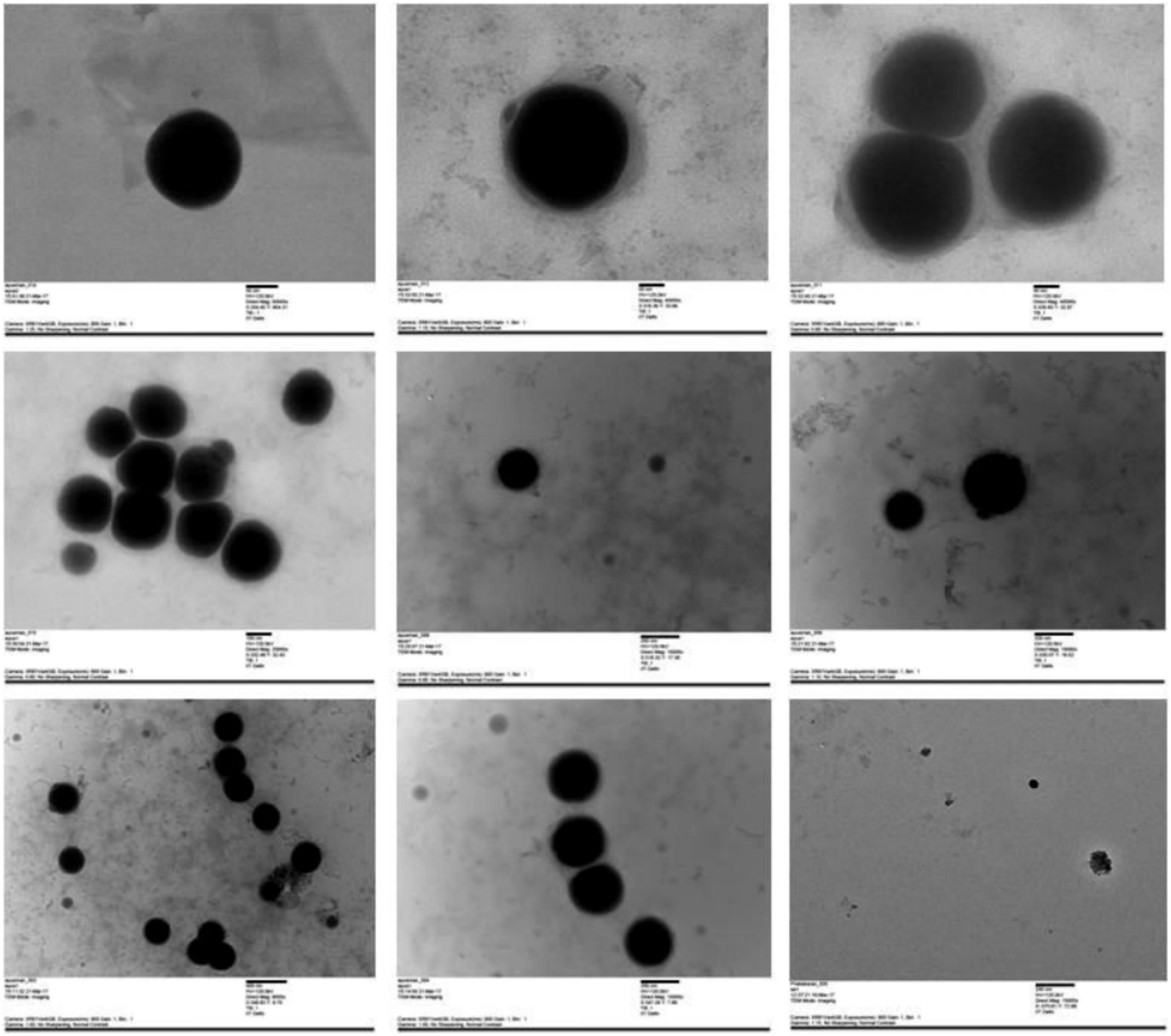

Fig S2. Transmission electron microscopic (TEM) images of Trichogenic-SeNPs of different *Trichoderma* used in the present study.

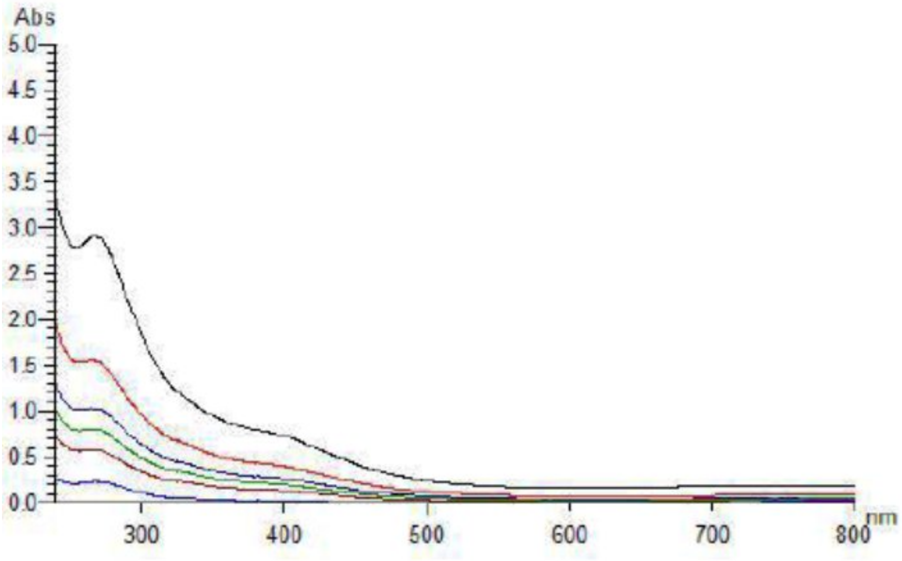

Fig S3. UV-Visible spectrum of CL-SeNPs showing an additional peak absorption peak at 415 nm.

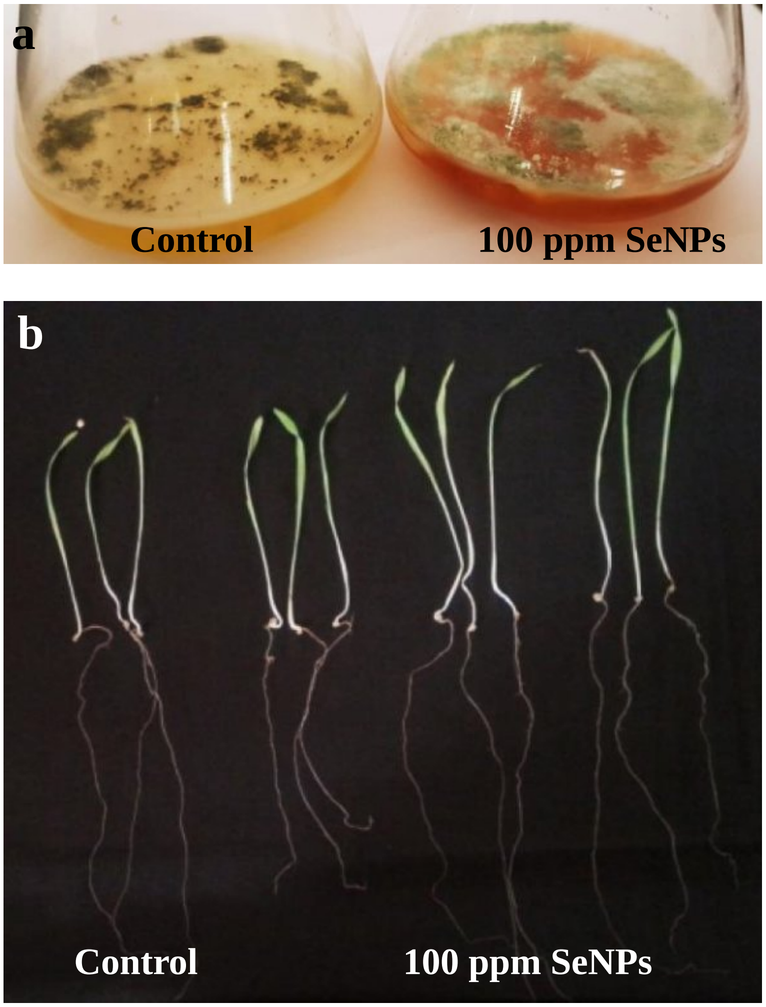

Fig S4. Effect of Trichogenic-SeNPs on the growth of (a) *T. asperellum* (b) pearl millet (PM) at 100 ppm concentration SeNPs.

Table S1. Anti-mildew activity of Trichogenic-selenium nanoparticles (SeNPs)

SeNPs	Concentration (ppm)																
	0	50	100	150	200	250	300	350	400	450	500	550	600	650	700	750	800
**Culture filtrate**																	
T.as-CF		C	B	A	A	A	A	A	A	A	A	A	A	A	A	A	A
T.ha-CF		D	D	D	D	C	B	B	A	A	A	A	A	A	A	A	A
T.vi-CF		D	D	C	C	B	B	A	A	A	A	A	A	A	A	A	A
T.lo-CF		D	C	C	B	A	A	A	A	A	A	A	A	A	A	A	A
T.at-CF		D	D	C	C	B	B	A	A	A	A	A	A	A	A	A	A
T.br-CF		D	D	C	B	A	A	A	A	A	A	A	A	A	A	A	A
**Cell lysate**																	
T.as-CL		D	D	D	C	B	B	A	A	A	A	A	A	A	A	A	A
T.ha-CL		D	D	D	D	D	C	C	C	C	B	B	A	A	A	A	A
T.vi-CL		D	D	C	C	C	C	B	B	B	A	A	A	A	A	A	A
T.lo-CL		D	D	D	D	C	C	B	B	B	A	A	A	A	A	A	A
T.at-CL		D	D	D	D	D	C	C	C	B	B	A	A	A	A	A	A
T.br-CL		D	D	D	D	C	C	C	B	B	B	A	A	A	A	A	A
**Crude cell wall**																	
T.as-CW		D	D	D	D	C	C	C	B	B	A	A	A	A	A	A	A
T.ha-CW		D	C	C	B	B	B	A	A	A	A	A	A	A	A	A	A
T.vi-CW		D	D	D	D	D	D	C	C	B	B	A	A	A	A	A	A
T.lo-CW		D	D	D	D	D	C	C	B	B	B	A	A	A	A	A	A
T.at-CW		D	D	C	C	B	B	A	A	A	A	A	A	A	A	A	A
T.br-CW		D	D	D	D	D	D	D	C	B	B	A	A	A	A	A	A

T.as - *T. asperellum*; T.ha – *T. harzianum*; T.vi – *T. virens*; T.lo – *T. longibrachiatum*; T.at – *T. atroviride*; T.br -

CF - Culture filtrate, CL - cell lysate; CW - crude cell wall.

Inhibition scale: A (100% inhibition), B (<100-75> % inhibition), C (<75-50> % inhibition), D (<50-25> % inhi
00> % inhibition)
